# Surgical and Anatomic Consideration in Endoscopic Dacryocystorhinostomy of a Patient with Damaged Sinonasal Anatomy Post–Caldwell-Luc Surgery: A Case Report

**DOI:** 10.3390/medicina58010078

**Published:** 2022-01-05

**Authors:** Chia-Chen Hsu, Lung-Chi Lee, Bo-I Kuo, Che-Jui Lee, Fang-Yu Liu

**Affiliations:** 1 Department of Ophthalmology, Tri-Service General Hospital, National Defense Medical Center, Taipei 114, Taiwan; water097978kaohsiung9@gmail.com (C.-C.H.); kidday0205@gmail.com (L.-C.L.); 2 Department of Ophthalmology, National Taiwan University Hospital, Taipei 100, Taiwan; bikuo0514@gmail.com; 3 Department of Otolaryngology-Head and Neck Surgery, Tri-Service General Hospital, National Defense Medical Center, Taipei 114, Taiwan; a5110998@gmail.com

**Keywords:** radical antrostomy, epiphora, lacrimal apparatus surgery, nasolacrimal duct obstruction, case report

## Abstract

*Background:* The Caldwell-Luc (CL) procedure, an outdated operative procedure that is used to treat inflammatory sinus diseases, is rarely performed presently. However, physicians may encounter patients with a history of CL surgery who develop considerable postoperative changes that may lead to diagnostic confusion in imaging evaluation; increase the difficulty of future surgery, such as sinonasal surgery; and increase the incidence of future intraoperative complications. *Case summary*: A 67-year-old man with a surgical history of chronic sinusitis reported epiphora of the left eye for five years. Balloon dacryocystoplasty was attempted but failed. Endo-DCR (Endoscopic dacryocystorhinostomy) was indicated; however, preoperative CT (computed tomography) imaging and nasal endoscopic examination showed sinonasal anomalies and the loss of internal landmarks for localizing the lacrimal sac. Preoperative CT results indicated previous CL surgery. Endo-DCR was performed with the aid of nasal forceps and a 20-gauge vitreoretinal fiberoptic endoilluminator. A six-month follow-up revealed the complete resolution of symptoms and no signs of recurrence. *Conclusions*: Epiphora might be a delayed complication of the CL procedure. Before performing endo-DCR, ophthalmologists should be familiar with the sinonasal anatomy and carefully assess preoperative imaging to identify anatomical variations. Nasal forceps and transcanalicular illumination can assist in determining the precise location of the lacrimal sac during endo-DCR.

## 1. Introduction

Endoscopic dacryocystorhinostomy (endo-DCR) is indicated for the restoration of tear drainage when the lacrimal pathway obstruction is at or below the level of the lacrimal sac. Compared with external DCR, endo-DCR has comparable long-term outcome and the advantages of avoiding external skin incision, preserving the pumping function of the orbicularis muscle, and permitting access for the treatment of concomitant sinonasal problems simultaneously [1,2,3,4,5]. For ophthalmologists, performing endoscopic DCR surgery is difficult and requires familiarity with the anatomy of both the lacrimal outflow apparatus and the nose. The usual anatomic landmarks that are used as endo-DCR surgical guides include the maxillary line (intranasal projection of the medial edge of frontal process of the maxilla), middle turbinate (MT), axilla of MT (the anterior most insertion of MT onto the maxilla), vertical plate of the uncinate process, and the bulla ethimoidalis [6]. However, these landmarks cannot be relied on at all times because anatomic structures may have variants or may change due to inflammation, neoplasm, congenital anomaly, surgery, or trauma.

The Caldwell-Luc (CL) approach, described originally by George Caldwell and Henri Luc more than a century ago, is the main procedure for approaching the maxillary sinus and nearby structures before the advocates of functional endoscopic sinus surgery (FESS) [7,8]. The operation involves an incision through the gingivobuccal mucosa sulcus, radical elimination of diseased maxillary content, and meatal antrostomy. Since the superiority of FESS to CL procedure in many aspects, such as better patient’s comfort, less intraoperative and postoperative hemorrhage, fewer perioperative complications, and shorter recovery time, the CL procedure is seldom performed today and only indicated for limited cases with maxillary sinus disease [9,10]. In addition, CL surgery could lead to profound postoperative changes of the sinonasal structures and increase difficulty of subsequent surgery [11,12]. 

Herein, we present a challenging case of nasolacrimal duct obstruction (NLDO) with a post-CL-procedure and the associated distorted intranasal anatomy receiving endo-DCR, and the evaluation of the preoperative assessment, computed tomography (CT) findings, and surgical techniques.

## 2. Case Report

A 67-year-old man presented with hypertension, diabetes mellitus, and a surgical history of left chronic sinusitis more than 50 years ago. Three years ago, he presented to our outpatient clinic because of tearing of his left eye for two years. According to medical records, a diagnostic probing test revealed a hard stop. Balloon dacryocystoplasty was performed but failed, and no further intervention was conducted due to loss to follow-up. This time, he returned with worsening epiphora and a new-onset yellowish mucopurulent discharge in the same eye.

An anterior segment assessment revealed a clear cornea with a high tear meniscus height in his left eye. There was no swelling of the medial canthus or abnormality of the eyelids, including lid laxity, ectropion, entropion, punctum eversion, and blepharitis. Lacrimal irrigation was performed; however, mucopurulent reflux through the upper punctum was noted. A dye disappearance test demonstrated delayed fluorescein clearance. Further diagnostic probing showed a hard stop, indicating the presence of a patent common canaliculus with an obstructed lacrimal sac or nasolacrimal duct. After discussing with the patient, he agreed to undergo endo-DCR surgery.

**Figure 1:**
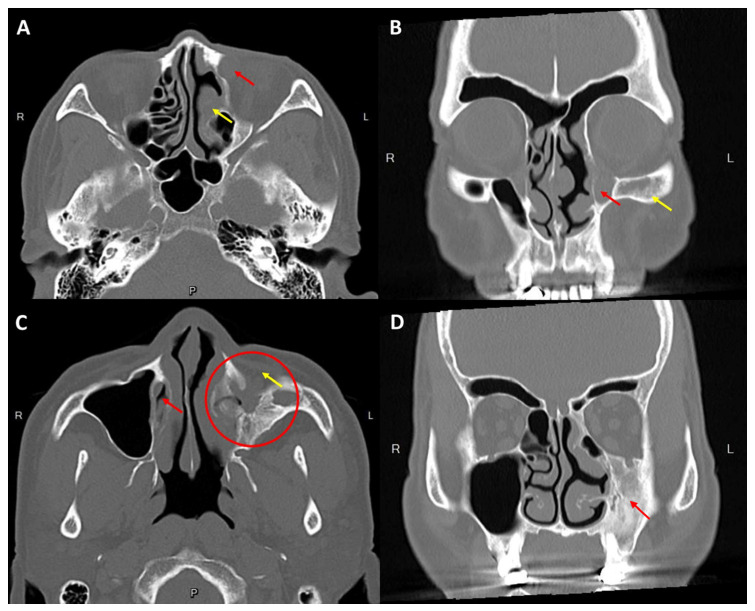
(**A**) Axial section through the lacrimal sac shows an expanded lacrimal sac (red arrow) and ethmoidectomy with superior turbinate hypertrophy on the left (yellow arrow). (**B**) Coronal computed tomography (CT) scan shows a prominent dilated intraosseous part of the left nasolacrimal fluid-filled duct (red arrow) and osseous proliferation of the left maxillary sinus (yellow arrow). (**C**) The lower axial section shows the intact terminus of the nasolacrimal canal on the right, within the inferior nasal meatus (red arrow), and totally obliterated on the left due to osseous proliferation and shrinkage of the left maxillary sinus (red circle). A bony defect of the anterior sinus wall (yellow arrow) was also observed. (**D**) The coronal CT scan shows osseous thickening, sinus retraction, and cavity volume reduction on the left maxillary sinus (red arrow) and increased volume of the left orbit.

As the patient had a surgical history of chronic sinusitis, a preoperative non-contrast computed tomography (CT) scanning (2-mm section) was performed to analyze the anatomical alterations. The imaging findings showed canine fossa bony defect, bony opening of the anterior and medial walls of the maxillary sinus, sclerosis, destruction and wall thickening of the maxillary sinus, and a collapse of the maxillary sinus cavity with distinct sinus volume reduction and increased orbital volume on the left. An expanded left lacrimal sac and a dilated nasolacrimal duct due to obliterated terminus were noted. Surgical landmarks, such as the maxillary line, axilla, uncinate process, and agger nasi, that are used to guide endo-DCR were absent. In addition, a minimally preserved MT was observed (Figure 1). A large structural defect and reactive bony change in the left maxillary sinus that was observed on CT indicated a possible previous CL surgery, which was compatible with the patient’s self-reported medical history.

**Figure 2:**
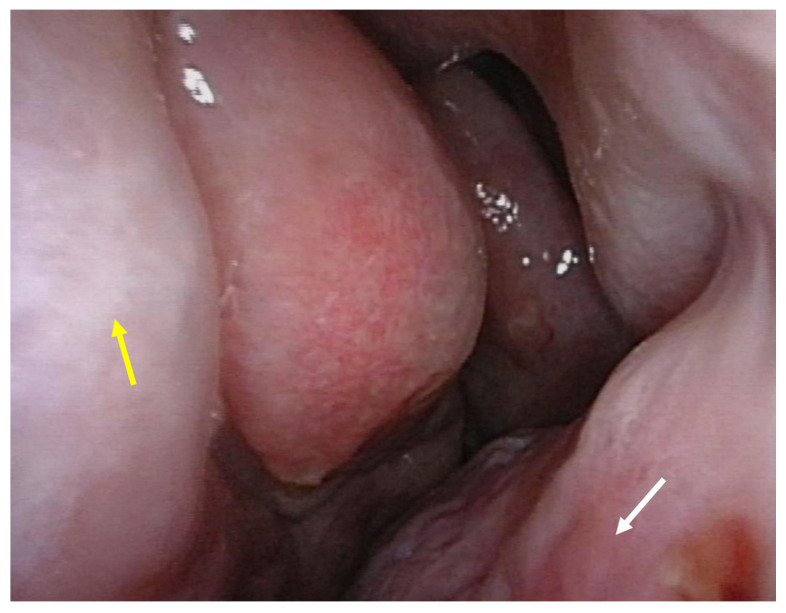
Preoperative nasal endoscopic examination. Polypoidal changes in the nasal septum (yellow arrow) and hypertrophy of inferior concha (white arrow) were noted. There is a lack of middle turbinate and axilla of middle turbinate.

The preoperative nasal endoscopic examination that was performed by a rhinologist showed polypoidal changes in the nasal septum, consolidation of the maxillary sinus, and enlarged space of the left nasal cavity due to the decreased size of the middle concha. No sinusitis was observed (Figure 2).

**Figure 3:**
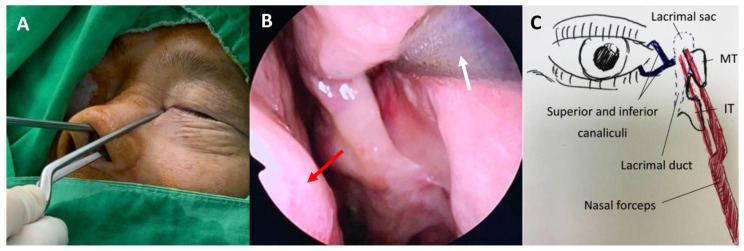
Intraoperative view of endoscopic dacryocystorhinostomy and illustration demonstrating the use of a Jansen bayonet nasal forceps to localize the lacrimal sac. (**A**) The outer tip of the forceps aligned with the medial canthus where the lacrimal sac was located. (**B**) The inner tip of the forceps pointed to the corresponding intranasal position of the lacrimal sac. The yellow arrow indicates the nasal septum, and the white arrow indicates the inner tip of the Jansen bayonet nasal forceps. (**C**) An illustration of lacrimal system anatomy showing the inner and outer tip of nasal forceps abut against the position of lacrimal sac. MT = middle turbinate; IT = inferior turbinate.

**Figure 4:**
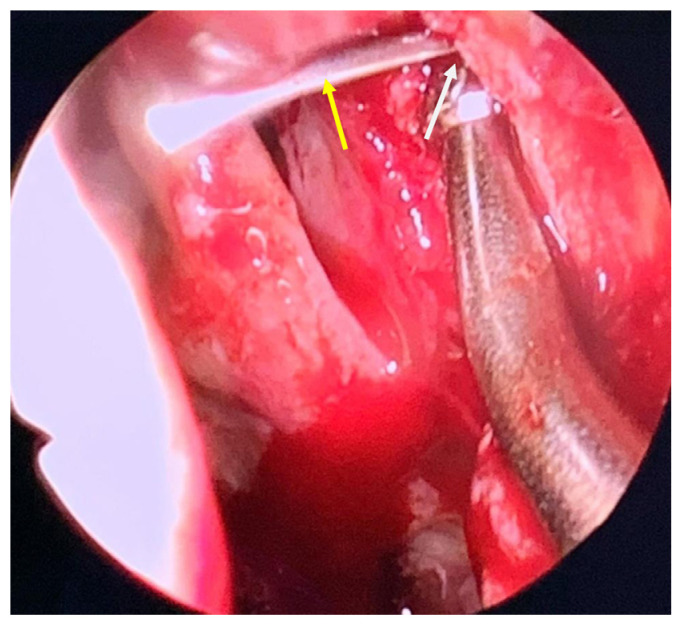
Intranasal view of opening of the lacrimal sac. A transcanalicular illuminator (yellow arrow) inserted through punctum being used to gain the location of lacrimal sac internally. The white arrow indicates the bony window.

The patient’s MT and axilla of the MT, which are the internal surface landmarks of the lacrimal sac, were absent. Thus, during the endo-DCR, we inserted nasal forceps (Jansen Bayonet Nasal/Ear Forceps^®^; Cross Instruments Inc., Edmonton, Canada) through both sides of the left nasal ala, with the outer tip of the forceps aligned with the medial canthus where the lacrimal sac was located. Subsequently, we lightly clipped together the inner tip, which then pointed to the corresponding intranasal position of the lacrimal sac (Figure 3A,B). After creating and raising a mucosal flap, the bone overlying the lacrimal sac was removed. With the aid of a 20-gauge transcanalicular light source, we observed the light through a nasal endoscope and exposed the lacrimal sac precisely by creating a large bony window of the lacrimal bone with a powered endoscopic diamond burr (Figure 4). The lacrimal sac was then marsupialized and a mucopurulent abscess was observed. Silicone stents (FCI Nunchaku^®^; FCI Ophthalmics, Pembroke, MA, USA) were inserted through the superior and inferior puncta into the nasal cavity. The whole procedure was performed smoothly without complications, and anatomical and functional success was achieved. A six-month follow-up revealed complete remission of the epiphora. The patient was quite satisfied with the surgical outcome as he was free from tearing without any postoperative external deformity.

## 3. Discussion

The CL procedure, also known as radical antrostomy, which was pioneered by George Caldwell and Henri Luc more than 100 years ago, is outdated [7,8]. In the era of FESS, the remaining indications for CL include maxillary sinus pathology (neoplasm and refractory inflammation), inverted papilloma, pterygopalatine fossa surgery, and orbital floor decompression [13]. The CL procedure involves entry through the canine fossa, removal of the diseased mucous membrane, and intranasal antrostomy, and sometimes, it is combined with ethmoidectomies. Although the CL procedure is not usually performed anymore, owing to its historic role, we may still encounter patients in the clinic who have undergone CL surgery. Postsurgical changes may be misinterpreted as traumatic deformity, congenital maxillary sinus hypoplasia, and malignancy-associated bony change and may lead to difficulty in further sinonasal surgery.

The maxillary sinus is the biggest paranasal sinus with a medial wall lying adjacent to nasal cavity and is the major site where the CL procedure is performed [14]. The changes in the maxillary sinus CT after the CL procedure have been described. The characteristic findings include bony defects in the anterior and medial sinus walls, osseous thickening, mucosal proliferation, sinus retraction, and cavity volume reduction [11,12]. In the present case, the CT scan revealed the typical image presentation that is mentioned above, except for inflammatory mucosal thickening of the left maxillary sinus. In addition, hypertrophy of the inferior concha, minimally preserved MT, orbital volume enlargement, and absence of the anterior ethmoid sinus were also observed in the left orbit and paranasal anatomy. Although our patient could not remember the type of sinonasal surgery he underwent, the characteristic image findings were deemed presumptive evidence of post-CL in conjunction with ethmoidectomies. Furthermore, a CT scan is also effective for evaluating the lacrimal drainage apparatus and identifying the possible etiology of NLDO. In this case, the obstruction of the lacrimal drainage system is associated with irreversible ossification and wall thickening of the maxillary sinus, leading to the obliteration of the inferior meatus where the nasolacrimal duct opens. Furthermore, the patient had no other predisposing factors for acquired NLDO, including facial or nasal trauma, topical medication, chemotherapy, radiation, autoimmune disease, and neoplasm. Thus, the NLDO in this patient was strongly associated with the previous CL procedure.

The CL procedure, although generally safe, is not without risk of complications. Recurrent sinusitis, facial and gum numbness/pain, oroantral fistula, and dental problems have been reported as complications of CL [13,15,16]. Epiphora following CL procedures predominantly occurs in the early postoperative periods due to edema-related functional blockage and spontaneously recovers in two to three months [17]. Only four cases of persistent NLDO post-CL procedure have been described, all of which were reported before 1991. In these cases, epiphora developed shortly after the surgery, usually within one week, resulting from iatrogenic injury to the opening of the nasolacrimal duct during inferior meatus antrostomy [18,19,20]. However, in the present case, epiphora developed more than 40 years after CL surgery. We surmised that the etiology of NLDO was not due to iatrogenic injury but was related to the long-term osseous proliferation of the maxillary sinus and relevant anatomy after surgery. Therefore, even though most of the complications of CL procedure are minor and temporary, permanent and late onset problems restrict the surgery a reserved therapy for rational indications.

Earlier literature suggested that external DCR is the procedure of choice for persistent NLDO post-CL procedure [18,19,20]. In this case, endo-DCR was performed, as it provides an excellent visualization of the intranasal structure and better cosmetic results. The nasolacrimal duct endonasally corresponds with the maxillary line lying on the lateral nasal wall. Nonetheless, a major part of the MT and the insertion site of the MT onto the maxill were damaged by the CL procedure in this case. When the usual anatomical landmarks for endo-DCR are absent, clipping Jansen bayonet nasal forceps through the bilateral side of the nasal ala can help in identifying the location of the lacrimal sac; by placing the outer tip at the medial canthus, the inner tip will point to the corresponding intranasal position overlying the lacrimal sac. We recognized that bayonet forceps, which are commonly used in otorhinolaryngology procedures and neurosurgery, are ideal for grasping tissue in small and deep spaces. This technique is simple, minimally invasive, does not require expensive instruments, and allows the precise positioning of the mucosal flap. Moreover, transcanalicular illumination was also used. The light reflex that was observed intranasally determines the target of the lacrimal sac. Either a 20-/23-gauge vitreoretinal fiberoptic endoilluminator or a lighted lacrimal stent could be inserted and this procedure could be conducted at the beginning of endo-DCR before the mucosa is incised [21,22,23]. However, in our experience, the light could be insufficient in the case of a very thick frontal process of the maxilla or hypertrophied nasal mucosa. Under such conditions, bayonet forceps can help in identifying the internal location of the sac. In addition to these useful methods, combined sinonasal surgery with otolaryngologists is also a preferred choice in complicated cases.

## 4. Conclusions

We reported a case of NLDO with characteristic CT findings after the CL procedure. The preoperative evaluation with CT is beneficial for identifying the anatomic variants, making appropriate assessments, and preparing precise surgical plans, and, thus, reducing possible complication rates. The combined use of nasal forceps and lacrimal illuminator allows a quick and easy way to localize the lacrimal sac during endo-DCR in cases of sinonasal anomalies. Surgical success and complete resolution of epiphora symptoms were achieved.

## Data Availability

The data presented in this study are available on request from the corresponding author. The data are not publicly available due to privacy.
